# Editorial: The role of transcription factors in inborn errors of immunity

**DOI:** 10.3389/fimmu.2023.1189312

**Published:** 2023-03-27

**Authors:** Delfien J. Bogaert, Hye Sun Kuehn, Victoria Bordon, Filomeen Haerynck

**Affiliations:** ^1^ Department of Pediatrics, Division of Pediatric Hemato-Oncology and Stem Cell Transplantation, Ghent University Hospital, Ghent, Belgium; ^2^ Department of Internal Medicine and Pediatrics, Primary Immunodeficiency (PID) Research Laboratory, Center for Primary Immunodeficiency Ghent, Jeffrey Modell Diagnosis and Research Center, Ghent University, Ghent, Belgium; ^3^ Immunology Service, Department of Laboratory Medicine, National Institutes of Health, Clinical Center, Bethesda, MD, United States; ^4^ Department of Pediatrics, Division of Pediatric Pulmonology, Immunology and Infectious Diseases, Ghent University Hospital, Ghent, Belgium

**Keywords:** transcription factor, inborn errors of immunity, AIOLOS, GATA2, IKAROS, KLF2, NFKB1, STAT5b

Inborn errors of immunity (IEI), also known as primary immunodeficiencies (PID), are a heterogeneous group of disorders caused by germline-encoded defects in the immune system resulting in an increased susceptibility to infections, autoimmune diseases, autoinflammation, benign lymphoproliferation, severe allergy, malignancies, and/or bone marrow failure. By use of advanced next-generation sequencing technologies, more than 485 IEI-associated genes have been reported to date. Pathogenic variants in transcription factors (TFs) have been identified in nearly all categories of IEI, emphasizing their widespread role across immune responses (1). TFs are key cellular proteins that bind to specific regulatory DNA motifs and modulate target gene expression and protein synthesis (2). The first fundamental insights in transcriptional regulatory mechanisms were established by Jacob and Monod in 1961 (3). Since then, alterations in transcriptional control have been shown to contribute to the development of many different diseases, including immune-related disorders and malignancies (2). In the field of IEI, the dynamic behavior of TFs in binding to DNA motifs, interacting with other proteins, and ultimately gene expression is a challenging subject (4, 5). The original research and review articles in this Research Topic illustrate the complexity of the genetic, immunological and clinical features of IEI caused by defects in TFs. Moreover, this article collection radiates the scientific effort that is being done worldwide to further elucidate the role of TFs in IEI.

Kuijpers et al. reported three patients from the same family carrying a novel, autosomal dominant variant in *IKZF1* causing IKAROS deficiency. The variant was predicted to result in a dimerization defect of the mutant IKAROS protein. IKAROS, encoded by *IKZF1*, is a hematopoietic zinc finger transcription factor important in lymphocyte development and differentiation. Somatic *IKZF1* alterations have been frequently documented in human leukemia, particularly in B cell acute lymphoblastic leukemia (B-ALL), in which they have been associated with an adverse impact on prognosis. Since 2016, heterozygous germline variants in *IKZF1* have been recognized to cause IEI, and can be classified into four categories depending on the mechanism of action (loss-of-function [haploinsufficiency, dimerization-defective, dominant-negative] and gain-of-function). Each category is characterized by a particular, though variable, clinical and immunological phenotype (5). The index patient in the report by Kuijpers et al. was diagnosed with common variable immunodeficiency (CVID), whereas the father was found to have asymptomatic selective IgA deficiency and the brother suffered from chronic idiopathic thrombocytopenia (ITP). This study is a clear example of the phenotypic diversity within a single family carrying the same genetic defect. We previously made a similar observation in a family with IKAROS haploinsufficiency (6). In these patients, the contribution of additional germline DNA alterations (modifier genes) and/or epigenetic modifications remains to be elucidated (7). Of interest, the authors also underline the diagnostic and therapeutic challenges that practitioners encounter in these patients.

IKAROS is one of five members of the IKAROS family of zinc finger transcription factors, all playing essential roles in hematopoiesis. Very recently, heterozygous germline variants in *IKZF2* (encoding HELIOS) and *IKZF3* (encoding AIOLOS) have also been associated with IEI, showing overlapping features with *IKZF1*-associated IEI (8–10). In this Research Topic, Yamashita and Morio published a comprehensive review on AIOLOS-associated IEI. Currently, two families with heterozygous loss-of-function *IKZF3* variants have been described. One variant (G159R) was characterized by B cell lymphopenia and an increased susceptibility to EBV-associated lymphoma. The other variant (N160S) resulted in a combined immunodeficiency (CID) phenotype including hypogammaglobulinemia and opportunistic infections, as well as an increased risk of chronic lymphocytic leukemia (CLL) (9, 10). Yamashita and Morio have clarified how elaborate studies of these variants have revealed the pivotal role of AIOLOS in T and B cell development and adaptive immune regulation not only in humans, but also in mice models.

The review by Fabozzi et al. focused on the genetics, clinical features and treatment options of guanine-adenine-thymine-adenine 2 (GATA2) deficiency. Similar to the IKAROS family of proteins, GATA2 is a zinc finger transcription factor crucial in hematopoiesis. More specifically, GATA2 regulates the development, self-renewal and expansion of hematopoietic stem cells (HSC). Germline heterozygous loss-of-function variants in *GATA2* were first reported in 2011, causing a complex phenotype involving hematological and immunodeficiency features, as well as lymphedema, sensorineural deafness, miscarriage, and pulmonary alveolar proteinosis. The age of onset and disease severity is very variable, even within the same family (11–14). Nowadays, GATA2 deficiency is mainly regarded as an inherited bone marrow failure disorder due to progressive depletion of the HSC pool (15). GATA2 deficiency is also considered one of the most frequent cancer predisposition syndromes for myeloid neoplasms, including myelodysplastic syndromes (MDS), especially in childhood (16). There are no consensus guidelines on the management of GATA2 deficient patients. The only curative treatment is allogeneic hematopoietic stem cell transplantation (HSCT). However, the optimal timing for HSCT, donor type and conditioning regimen remain unclear (17). In this review, the authors discussed the state-of-the-art of GATA2 deficiency, pinpointing the challenges and opportunities for future research.

Smith et al. provided a comprehensive review on the biology and role in human disease of the two Signal Transducer and Activator of Transcription (STAT) 5 paralogs, STAT5a and STAT5b. The STAT5 proteins are key transcription factors in numerous biological processes, including hematopoiesis and immunity. STAT5 signaling is activated by a variety of cytokines, hematopoietic growth factors, and growth hormone. Both somatic and germline variants in *STAT5* have been associated with immune-related diseases and malignancies in humans (18). In particular, germline variants in *STAT5B* causing STAT5b deficiency are associated with IEI (19). Smith et al. dedicated an important section of their review on the latter, covering the wide spectrum of clinical manifestations seen in these rare patients. Furthermore, the authors elaborated on the molecular mechanisms of STAT5 alterations in human disease, their contributions in several types of cancer, and options for targeted therapy. Overall, this review is a nice illustration of the complexity of JAK/STAT signaling in immune and non-immune cells, and how the multilevel regulation of these proteins and the dynamics of countless protein-DNA and protein-protein interactions complicate research efforts as well as clinical practice.

Nuclear factor of κ light polypeptide gene enhancer in B cells 1 (NF-κB1) deficiency, caused by heterozygous loss-of-function variants in *NFKB1*, is one of the most frequent monogenic subtypes of CVID in Europe and North America (20). NF-κB1 is one of the five transcription factors of the (NF-κB)/Rel family, and plays a pivotal role in inflammatory and immune responses. Defects in NF-κB1 signaling have not only been associated with IEI, but also autoimmunity and cancer (21) (Barnabei et al.). Upon activation of NF-κB1, the precursor protein p105 is proteolytically processed into the subunit p50 which translocates to the nucleus in p50-containing dimers to exert its transcriptional activity. The work of Fliegauf et al. investigated a functional validation method for missense variants in *NFKB1* located in the N-terminal domains, thereby affecting both p105 and p50. The authors analyzed 47 missense variants and were able to demonstrate deleterious loss-of-function defects in about half of them. This study will facilitate the functional validation of *NFKB1* missense variants in the future. On the other hand, for many missense variants a possible pathogenic or benign impact could not be determined, underlining the difficulty of interpretating such variants and the need for additional functional validation assays.

Finally, Pernaa et al. described a kindred with a novel IEI caused by an autosomal dominant variant in Krüppel-like factor 2 (KLF2). KLF2 is a zinc finger transcription factor expressed in several cell types, but is especially important in endothelial cells, lymphocytes, monocytes, and adipocytes. KLF2 also exerts a regulatory effect on the NF-κB signaling pathway (22, 23). The heterozygous frameshift variant identified by Pernaa et al. disrupted the conserved zinc finger domain of KLF2 resulting in defective protein activity. The corresponding phenotype encompassed T and B cell lymphopenia, maturation abnormalities in T and B cells, normal to mildly reduced immunoglobulins, (respiratory tract) infections, autoimmune diseases, and malignancies. The KLF2 variant carriers showed variable expressivity and incomplete penetrance, similar to what is seen in many other heterozygous IEI disorders (1). Identification of additional patients with KLF2 variants will be required to further unravel the genotypical and phenotypical spectrum of this new IEI entity.

Together, the publications collected in this Research Topic highlight the critical role of TFs in regulating immune cell development and differentiation. These articles are useful to the reader in understanding the multilevel complexity by which defects in TFs can result in IEI with diverse clinical pictures including immune dysregulation and malignancies. Moreover, this Research Topic will assist researchers in selecting appropriate assays to functionally validate variants of unknown significance.

## Author contributions

All authors listed have made a substantial, direct and intellectual contribution to the realization of the Research Topic and editorial manuscript, and approved the final manuscript as submitted.

## References

[1] Tangye SG Al-Herz W Bousfiha A Cunningham-Rundles C Franco JL Holland SM. Human inborn errors of immunity: 2022 update on the classification from the international union of immunological societies expert committee. J Clin Immunol (2022) 42:1473–1507. doi: 10.1007/s10875-022-01289-3 PMC9244088 35748970

[2] Lee TI Young RA. Transcriptional regulation and its misregulation in disease. Cell (2013) 152:1237–51. doi: 10.1016/j.cell.2013.02.014 PMC3640494 23498934

[3] Jacob F Monod J. Genetic regulatory mechanisms in the synthesis of proteins. J Mol Biol (1961) 3:318–56. doi: 10.1016/S0022-2836(61)80072-7 13718526

[4] Vogel TP Milner JD Cooper MA. The ying and yang of STAT3 in human disease. J Clin Immunol (2015) 35:615–23. doi: 10.1007/s10875-015-0187-8 PMC4628878 26280891

[5] Kuehn HS Boast B Rosenzweig SD. Inborn errors of human IKAROS: LOF and GOF variants associated with primary immunodeficiency. Clin Exp Immunol (2022). doi: 10.1093/cei/uxac109 PMC10128159 36433803

[6] Bogaert DJ Kuehn HS Bonroy C Calvo KR Dehoorne J Vanlander AV. A novel IKAROS haploinsufficiency kindred with unexpectedly late and variable b-cell maturation defects. J Allergy Clin Immunol (2018) 141:432–35.e7. doi: 10.1016/j.jaci.2017.08.019 28927821 PMC6588539

[7] Bogaert DJ Dullaers M Lambrecht BN Vermaelen KY De Baere E Haerynck F. Genes associated with common variable immunodeficiency: one diagnosis to rule them all? J Med Genet (2016) 53:575–90. doi: 10.1136/jmedgenet-2015-103690 27250108

[8] Shahin T Mayr D Shoeb MR Kuehn HS Hoeger B Giuliani S. Identification of germline monoallelic mutations in IKZF2 in patients with immune dysregulation. Blood Adv (2022) 6:2444–51. doi: 10.1182/bloodadvances.2021006367 PMC9006292 34920454

[9] Yamashita M Kuehn HS Okuyama K Okada S Inoue Y Mitsuiki N. A variant in human AIOLOS impairs adaptive immunity by interfering with IKAROS. Nat Immunol (2021) 22:893–903. doi: 10.1038/s41590-021-00951-z 34155405 PMC8958960

[10] Kuehn HS Chang J Yamashita M Niemela JE Zou C Okuyama K. T And b cell abnormalities, pneumocystis pneumonia, and chronic lymphocytic leukemia associated with an AIOLOS defect in patients. J Exp Med (2021) 218:e20211118. doi: 10.1084/jem.20211118 34694366 PMC8548914

[11] Hsu AP Sampaio EP Khan J Calvo KR Lemieux JE Patel SY. Mutations in GATA2 are associated with the autosomal dominant and sporadic monocytopenia and mycobacterial infection (MonoMAC) syndrome. Blood (2011) 118:2653–5. doi: 10.1182/blood-2011-05-356352 PMC3172785 21670465

[12] Hahn CN Chong CE Carmichael CL Wilkins EJ Brautigan PJ Li XC. Heritable GATA2 mutations associated with familial myelodysplastic syndrome and acute myeloid leukemia. Nat Genet (2011) 43:1012–7. doi: 10.1038/ng.913 PMC3184204 21892162

[13] Dickinson RE Griffin H Bigley V Reynard LN Hussain R Haniffa M. Exome sequencing identifies GATA-2 mutation as the cause of dendritic cell, monocyte, b and NK lymphoid deficiency. Blood (2011) 118:2656–8. doi: 10.1182/blood-2011-06-360313 PMC5137783 21765025

[14] Ostergaard P Simpson MA Connell FC Steward CG Brice G Woollard WJ. Mutations in GATA2 cause primary lymphedema associated with a predisposition to acute myeloid leukemia (Emberger syndrome). Nat Genet (2011) 43:929–31. doi: 10.1038/ng.923 21892158

[15] McReynolds LJ Calvo KR Holland SM. Germline GATA2 mutation and bone marrow failure. Hematology/oncol Clinics North America (2018) 32:713–28. doi: 10.1016/j.hoc.2018.04.004 PMC6128284 30047422

[16] Bruzzese A Leardini D Masetti R Strocchio L Girardi K Algeri M. GATA2 related conditions and predisposition to pediatric myelodysplastic syndromes. Cancers (2020) 12:2962. doi: 10.3390/cancers12102962 PMC7602110 33066218

[17] Bogaert DJ Laureys G Naesens L Mazure D De Bruyne M Hsu AP. GATA2 deficiency and haematopoietic stem cell transplantation: challenges for the clinical practitioner. Br J Haematol (2020) 188:768–73. doi: 10.1111/bjh.16247 31710708

[18] Kim U Shin HY. Genomic mutations of the STAT5 transcription factor are associated with human cancer and immune diseases. Int J Mol Sci (2022) 23:11297. doi: 10.3390/ijms231911297 PMC9569778 36232600

[19] Hwa V. STAT5B deficiency: Impacts on human growth and immunity. Growth Horm. IGF Res (2016) 28:16–20. doi: 10.1016/j.ghir.2015.12.006 26703237 PMC4846566

[20] Li J Lei WT Zhang P Rapaport F Seeleuthner Y Lyu B. Biochemically deleterious human NFKB1 variants underlie an autosomal dominant form of common variable immunodeficiency. J Exp Med (2021) 218:e20210566. doi: 10.1084/jem.20210566 34473196 PMC8421261

[21] Concetti J Wilson CL. NFKB1 and cancer: Friend or foe? Cells (2018) 7:133. doi: 10.3390/cells7090133 30205516 PMC6162711

[22] McConnell BB Yang VW. Mammalian krüppel-like factors in health and diseases. Physiol Rev (2010) 90:1337–81. doi: 10.1152/physrev.00058.2009 PMC2975554 20959618

[23] Wittner J Schuh W. Krüppel-like factor 2 (KLF2) in immune cell migration. Vaccines 9 (2021) 1171. doi: 10.3390/vaccines9101171 PMC8539188 34696279

